# Recruitment and Retention of Pregnant Women for a Behavioral Intervention: Lessons from the Maternal Adiposity, Metabolism, and Stress (MAMAS) Study

**DOI:** 10.5888/pcd10.120096

**Published:** 2013-03-07

**Authors:** Kimberly Coleman-Phox, Barbara A. Laraia, Nancy Adler, Cassandra Vieten, Melanie Thomas, Elissa Epel

**Affiliations:** Author Affiliations: Barbara A. Laraia, University of California, San Francisco, California, and University of California, Berkeley, California; Nancy Adler, Melanie Thomas, Elissa Epel, University of California, San Francisco, California; Cassandra Vieten, California Pacific Medical Center Research Institute, San Francisco, California.

## Abstract

**Introduction:**

Recruiting participants for research studies can be challenging. Many studies fall short of their target or must prolong recruitment to reach it. We examined recruitment and retention strategies and report lessons learned in a behavioral intervention developmental trial to encourage healthy pregnancy weight gain and stress reduction in low-income overweight pregnant women.

**Methods:**

In the San Francisco Bay area from February 2010 through March 2011, we used direct and indirect strategies to recruit English-speaking overweight and obese pregnant women who were aged 18 to 45, were in the early stages of pregnancy, and who had an annual household income less than 500% of the federal poverty guidelines. Eligible women who consented participated in focus groups or an 8-week behavioral intervention. We identified successful recruiting strategies and sites and calculated the percentage of women who were enrolled and retained.

**Results:**

Of 127 women screened for focus group participation, 69 were eligible and enrolled. A total of 57 women participated in 9 focus groups and 3 women completed individual interviews for a completion rate of 87%. During recruitment for the intervention, we made contact with 204 women; 135 were screened, 33% were eligible, and 69.1% of eligible women enrolled. At 1 month postpartum, 82.6% of eligible women completed an assessment. Recruiting at hospital-based prenatal clinics was the highest-yielding strategy.

**Conclusion:**

The narrow window of eligibility for enrolling early stage pregnant women in a group intervention presents obstacles. In-person recruitment was the most successful strategy; establishing close relationships with providers, clinic staff, social service providers, and study participants was essential to successful recruitment and retention.

## Introduction

Recruiting participants for research studies can be challenging; many studies fall short of their targeted sample size or must prolong recruitment to reach it (1). Recruitment and retention of low-income urban residents is particularly challenging and may be complicated by lack of time, transportation, and child care; inflexible work schedules; unstable housing and unsafe neighborhood conditions; chaotic lives; and distrust of medical institutions and research (2). Studies with narrow eligibility criteria (3) or complex protocols face additional obstacles (4). Adequate sample recruitment is essential to conduct high-quality studies. We examined recruitment and retention strategies used in the Maternal Adiposity, Metabolism, and Stress (MAMAS) study (National Institutes of Health [NIH], ClinicalTrials.gov identifier no. NCT01307683), a behavioral intervention development trial to encourage healthy pregnancy weight gain and stress reduction in low-income overweight and obese pregnant women.

Nearly 42% of US women enter pregnancy overweight or obese (5) and 60% exceed Institute of Medicine (IOM) gestational weight gain (GWG) recommendations (6). Prevalence of overweight and obesity is especially high among African American and Hispanic women of childbearing age (7). Low-income women are at additional risk for obesity because residence in poor neighborhoods may limit access to nutritious food and opportunities for physical activity (8,9). Excess GWG increases the risk for pregnancy complications including hypertension, preeclampsia, gestational diabetes, and cesarean birth (10,11). Infants born to overweight and obese women are at increased risk for neural tube defects (12), large for gestational age (13), childhood obesity (14), and type 2 diabetes in adulthood (15). Although clear IOM GWG guidelines exist (16), no standard of care protocol exists to help women achieve optimal GWG. Therefore, it is essential to target this high-risk group and recruit low-income women who are obese and in early pregnancy. 

The objective of the MAMAS study is to assess interest in and test feasibility of behavioral interventions to reduce stress-induced nonhomeostatic eating (eating in response to factors other than hunger or caloric need) during pregnancy by decreasing stress and increasing awareness of hunger, satiety, and automatic eating patterns. In this article we report screening, eligibility, enrollment, and retention rates during Phases 1 and 2 of the MAMAS study and identify facilitators of and barriers to participant recruitment and retention. 

## Methods

### Study design

The MAMAS study is funded by an NIH U01 mechanism that facilitates developmental approaches to translating basic research into effective interventions. In Phase 1, we conducted a qualitative study using focus groups with overweight and obese pregnant women to assess interest in and identify potential barriers to participating in a prenatal behavioral intervention. In Phase 2, we conducted an exploratory study to compare the effects of 2 promising group interventions, Emotional Brain Training (EBT) and a mindfulness-based intervention (MIND), on feasibility, acceptability, attendance and retention rates, stress, and weight gain. EBT uses cognitive behavioral and attachment theory to increase self-regulation of emotions and eating behavior (17). MIND is adapted from The Mindful Motherhood Training (18) and Mindfulness-Based Eating Awareness Training (19) and incorporates components from other mindfulness-based interventions (20–22). In addition to attending 8 weekly 2-hour intervention classes, participants were asked to complete questionnaires and clinical assessments at baseline, postintervention, and postpartum. Phases 1 and 2 were conducted in the San Francisco Bay area from February 2010 through March 2011. In Phase 3 (under way), we are conducting a clinical trial comparing the chosen intervention with usual care, which varies by provider. Study procedures were approved by the University of California, San Francisco, Committee on Human Research and the California Pacific Medical Center Institutional Review Board.

### Context and target population

Pregnancy is a time of emotional and physical changes when women may be more open to making lifestyle changes to improve their health and the health of their unborn child. This time may be considered a key “teachable moment” (23). Given high rates of overweight and obesity and levels of stress among low-income women, we sought to enroll English-speaking overweight and obese pregnant women (prepregnancy body mass index [BMI] 25.0–40.0 kg/m^2^), aged 18 to 45 with an annual household income less than 500% of the federal poverty guidelines ($72,850 for a family of 2) (24). Our recruitment goal was 50 to 100 focus group participants in Phase 1 and 48 intervention participants in Phase 2. During Phase 2, enrollment was limited to women at less than 20 weeks’ gestation so that they would receive the intervention early enough in pregnancy to affect their GWG trajectory. Because second trimester pregnancy loss is rare (25), we selected 12 weeks as the minimum gestational age at enrollment. The gestational age at entry to prenatal care was typically 7 to 14 weeks in our population. We aimed to enroll a cohort of 12 to 15 pregnant women in each intervention class series to obtain a sample of 48 women who would complete the intervention by 20 to 28 weeks’ gestational age.

Our recruitment strategy was twofold: we directly targeted pregnant women and the people and organizations who serve them. During Phase 1, we contacted various prenatal health care providers; county program managers with Maternal, Child, and Adolescent Health and the Special Supplemental Nutrition Program for Women, Infants, and Children (WIC); and other social services agencies to gather information about the characteristics and numbers of people served and to inform them about the study. We also requested recommendations for other service providers for low-income women and children and contacted recommended providers. Subsequently, we mailed recruitment packets containing flyers with tear-off tabs and “frequently asked questions” handouts to more than 200 providers who served our target population. We asked organizations with electronic mailing lists to send study announcements. We posted study flyers at neighborhood venues frequented by pregnant women (eg, laundromats, grocery stores, resale stores, and community centers). To increase awareness in the academic community, study investigators presented the study at grand rounds, didactic sessions for clinical residents, clinical staff meetings, and other regional meetings. After Phase 1, we sent tailored thank-you letters to referring providers, indicating the total number of focus group participants enrolled and the provider-specific number of women referred and enrolled. Focus group participants received assistance with transportation, parking, and child care costs and a $50 grocery or department store gift cards as compensation.

During Phase 2, we continued using the strategies used during Phase 1 and also recruited pregnant women at 3 hospital-based prenatal clinics that offered Comprehensive Perinatal Services Programs (CPSP). CPSP provides services to Medicaid-eligible women including prenatal care, health education, nutrition services, and psychosocial support. In communications with prenatal care providers, we described the study as a resource for overweight and obese patients and developed site-specific recruitment procedures in collaboration with clinic staff. To thank and reinforce clinic staff for their help in recruiting, we provided a healthful breakfast for them every 1 to 2 months. Two to 3 weeks before the start of each intervention series, a study investigator, who is a practicing obstetrician, e-mailed a reminder with the intervention start date and eligibility criteria to more than 300 obstetrics and gynecology clinical and administrative staff. We also mailed reminder letters containing small tokens (ie, pens or notepads with the study logo) to all potential recruitment sources. Following each intervention series, we mailed thank-you notes to referring organizations and offered to provide on-site stress reduction trainings for staff at organizations that referred enrolled women.

Recruitment was conducted in sequential waves to identify cohorts of eligible women in Phase 2. Assignment to intervention groups was initially by cohort (wave 1); the first cohort of eligible women was assigned to EBT and the second was assigned to MIND. When recruitment numbers increased (wave 2), eligible women were assigned by using random numbers to attend either EBT or MIND. Recruitment and assessment staff members were blinded to group assignment.

In Phase 2, to enhance retention and informed consent, eligible women were asked before enrollment to attend a group orientation, which included a brief introduction to research methods, a detailed explanation of study procedures and timeline, and an interactive example of the effects of differential loss to follow-up. The orientation culminated in a discussion of participant-generated pros and cons of participation; the orientation facilitator assumed the role of active listener rather than attempting to counter perceived disadvantages raised by participants (26). Orientation participants received $10 for attending the session. Women unable to attend a group orientation met with research staff for individual orientation but were not paid. Although the required orientation was an additional hurdle for potential enrollees, it made clear the commitment expected of participants and the demands of participation.

To enhance retention among enrolled participants, we collected all available contact information from participants (eg, mailing and e-mail addresses, home and cellular telephone numbers), mailed appointment reminder letters, and provided reminder telephone calls and e-mails. We also requested contact information for 2 people not living with the participant and, with participant consent, mailed letters to these additional contacts to introduce the study and advise them of future contact if we lost contact with the participant. Compensation for participation ranged from $50 to $275 in VISA gift cards, depending on the study components completed. Women also received $25 in cash at each intervention class to assist with transportation and child care costs.

Beginning at 35 weeks’ gestation, we called participants or their designated contact person weekly to determine when the participant gave birth. Our team sent personalized cards with the study logo to congratulate participants on the birth of their babies and remind them about the 1-month postpartum assessment. Participants received gift bags containing an infant “onesie” with the study logo, a compact disc of lullabies, and samples of baby toiletries at the postpartum assessment.

### Data collection and analysis

Screening data and recruitment source were collected using SurveyMonkey (Palo Alto, California). We tracked participants’ study progress using a Microsoft Access (Microsoft Corp, Redmond, Washington) database. We calculated eligibility, enrollment, and retention rates in both phases of the study. We compared retention rates by intervention type and study wave using χ^2^ tests to assess group differences. Analyses were conducted using Stata version 12.0 (StataCorp LP, College Station, Texas).

## Results

Of 127 women screened during Phase 1 (single focus group), 69 women were eligible and enrolled. Having a BMI of less than 25 kg/m^2^ (n = 46) was the most frequent reason for ineligibility, followed by having an annual household income higher than 500% of federal poverty guidelines (n = 5), having medical or psychosocial reasons (n = 3), being younger than age 18 (n = 2), speaking a language other than English (n = 1), and not being pregnant (n = 1). A total of 57 women participated in 9 focus groups, and 3 women completed individual interviews because of health restrictions, for a completion rate of 87%. The mean number of weeks of gestation for Phase 1 participants was 20.9 (standard deviation [SD], 9 weeks). Among the women who did not complete Phase 1, two gave birth early, 1 was unable to attend any groups because of scheduling conflicts, and 6 were lost to follow-up. Eligible participants learned about the study from hospital-based prenatal clinics (n = 17), social services providers (n = 11), prenatal programs (ie, Healthy Start, Black Infant Health) (n = 9), friends or relatives (n = 6), community health centers (n = 5), and WIC (n = 4) (Table).

**Table:** Recruitment Sources of Eligible Pregnant Women in the Maternal Adiposity, Metabolism, and Stress Study, San Francisco, California

Source	Phase 1 Focus Groups (February 1–May 28, 2010)			Phase 2 Intervention (September 1, 2010–March 14, 2011)		
	Total Contacted	Eligible	Participated	Total Contacted	Eligible	Participated
Hospital-based prenatal clinic	39	17	14	76	44	32
Hospital newsletter	15	5	4	12	3	2
Social services provider	13	11	11	12	4	3
Prenatal programs	13	9	9	8	3	1
Community health center	7	5	4	9	4	2
WIC	5	4	3	6	3	3
Friend or relative	5	6	6	11	3	1
Other	30	12	9	70	4	3
Total	127	69	60	204	68	47

Abbreviation: WIC, Special Supplemental Nutrition Program for Women, Infants, and Children.

During Phase 2 (8-week intervention), we contacted 204 women; 135 were screened, 68 were eligible, and 47 enrolled (Table). We did not screen women who disclosed at initial contact that they had low BMI, did not meet the gestational age criteria, had scheduling conflicts, or declined to answer screening questions. Nearly 24% of screened women were ineligible because of having a BMI less than 25 or higher than 40 (n = 32). Other reasons for exclusion included early (<12 weeks) or late (≥20 weeks) gestational age at the start of the intervention (n = 12), high income (n = 10), medical or psychosocial reasons (n = 7), age younger than 18 or older than 45 (n = 4), and logistical barriers to attending intervention sessions (n = 2). The mean number of weeks of gestation at enrollment was 17.6 (SD, 2.6 weeks). Almost 65% of eligible women were identified at hospital-based prenatal clinics in Phase 2.

Among the 47 enrolled participants in Phase 2, two women were lost to follow-up, 1 withdrew due to a scheduling conflict, and 1 was asked to withdraw because of disruptive behavior. The remaining 43 participants had mean attendance of 71.3% of all intervention classes. There was no significant difference in retention between the 2 interventions. Among EBT participants, 78.9% attended 4 or more classes compared with 75% of MIND participants (*P* = .76). In wave 1 of Phase 2, study participation ended with the postintervention questionnaire 1 to 2 weeks after the 8-week intervention, and 73.7% of participants completed the study. During the second wave of recruitment, when women were asked to attend an additional clinic assessment at 1 month postpartum, 19 (83%) of 23 eligible women completed the assessment (1 woman was ineligible because of fetal demise and 3 women were lost to follow-up). Differences between the 2 waves in rates of overall study retention were not significant (*P* = .52).

In postintervention evaluations, participants expressed high overall satisfaction with the program (89%) and thought the skills learned would be very helpful in managing weight during pregnancy and postpartum (75%). Additionally, they identified the opportunity to interact with other pregnant women in a group setting as an attractive and important feature (81%).

## Discussion

The percentage of eligible women who enrolled in the study was high; 100% of eligible women consented to participate in Phase 1, and 69% of eligible women consented to participate in Phase 2. Although our results are comparable to recruitment rates of pregnant women in other studies (27,28), we were particularly interested in exploring what factors were facilitators of and barriers to recruitment and retention.

### Facilitators of recruitment and retention

The wide network of medical and social services providers with whom we cultivated relationships was crucial to achieving our recruitment goals in Phase 1. In Phase 2, we relied on direct recruitment at hospital-based prenatal clinics to enroll most eligible participants, in addition to the passive strategies (eg, posting flyers, mailing information).

Phase 1 required attending only 1 group session, and scheduling focus groups at varied times and locations in the San Francisco Bay Area appeared to improve study completion. Hospitals near transit hubs and social services agencies permitted us to conduct focus groups at their sites. In addition to providing meeting space, many of the social services agencies with whom we partnered referred their clients, and 2 agencies provided transportation and child care for focus group participants.

Hospital-based prenatal care providers and clinic staff were very supportive of the study, but their clinical responsibilities left limited time to assist with patient recruitment. Moreover, the rotation of medical residents required study staff to frequently “recruit” new prenatal care providers as well as patients. At these sites, study personnel developed close working relationships with front-desk staff and medical assistants and relied on their help to identify potentially eligible patients. Building relationships with hospital-based prenatal clinic staff and performing on-site recruitment was a time-intensive but high-yielding strategy with 68% of all Phase 2 participants enrolled through direct recruitment. Direct recruitment is therefore the most critical recruitment strategy to communicate with and attract pregnant women in a narrow window for enrollment.

We retained nearly 74% of wave 1 participants at 2 weeks postintervention and 83% of wave 2 participants at 1 month postpartum. Retention rates in our study are similar to those achieved in the trials of El-Khorazaty and colleagues (2) (79% at 1 month postpartum) and Webb and colleagues (28) (83.5% at 8 to 10 weeks postpartum). Although we did not ask participants what influenced their decisions to return for the final assessment, we postulate that adequate financial incentives, orientation before enrollment, and multiple interactions with the same small research team helped to build rapport, promote trust, and instill a sense of commitment to the study.

### Barriers to recruitment and retention

Despite the positive response to our study, many factors affected our ability to achieve our targeted sample size in Phase 2. Inclusion criteria of 12 to 19 weeks’ gestation restricted the number of women who were eligible to enroll in a given period. Thus, during Phase 2, some otherwise eligible women were too early in pregnancy on the intervention start date, and an even larger number of women were excluded from participating because they were too late in pregnancy to benefit. Because the interventions were delivered in groups, the need to enroll a cohort of overweight pregnant women of similar gestational age (as opposed to individual women, 1 at a time) added another barrier to study entry. The short duration of pregnancy, the need for enrollment before 20 weeks’ gestation, and pregnancy losses in the interval between screening and the intervention start date reduced enrollment rates.

Logistical barriers common in low-income samples, such as inflexible work schedules, lack of transportation, reliance on public transit, and need for child care also negatively affected our ability to recruit low-income women. Schedule conflicts, transportation difficulties, and child care issues that were manageable for a 1-time focus group in Phase 1 in some cases became insurmountable obstacles during Phase 2 for women who wanted to attend an 8-week intervention. Although 89% of intervention participants reported being very satisfied with their experience, mean attendance of 73% of intervention sessions may reflect the difficulty of overcoming structural barriers to consistent participation.

Recruiting and retaining adequate numbers of participants in research studies is difficult. The narrow window of eligibility for enrolling women in a group intervention during pregnancy presents unique obstacles. Everyday demands of life (eg, child care, time, transportation) presented obstacles to enrollment for this low-income population. It was necessary to screen 3 pregnant women for each 1 enrolled. Given the labor-intensive process of recruiting a small cohort of overweight or obese pregnant women to participate in a feasibility trial, a large randomized clinical trial would likely require the participation of multiple sites to achieve an adequate sample size.

Of an array of direct and indirect recruitment strategies, we found that in-person recruitment at hospital-based prenatal clinics produced the highest yield of participants. Establishing close relationships with prenatal care providers, clinic staff, social service providers, and study participants was an essential precursor to successful recruitment and retention of our low-income study participants.
